# Ranking microbial risk of self-supplied groundwater in urban Indonesia

**DOI:** 10.1007/s10661-026-15909-7

**Published:** 2026-09-23

**Authors:** Paul Hansen, M. Adhiraga Pratama, Cindy Priadi, Dasapta Erwin Irawan, Tim Foster, Juliet Willetts

**Affiliations:** 1 Institute for Sustainable Futures, University of Technology Sydney, Sydney, Australia; 2 Department of Civil and Environmental Engineering, Faculty of Engineering, Universitas Indonesia, Kampus UI Depok, Depok, Indonesia; 3 Faculty of Earth Sciences and Technology, Institut Teknologi Bandung, Bandung, Indonesia

**Keywords:** *E. coli*, Groundwater self-supply, On-site sanitation, Urban hydrogeology, Private wells

## Abstract

In Indonesia, 61% of urban households use self-supplied groundwater, with 24% relying on it as a primary drinking water source. Many self-supplied sources contain faecal contamination, posing health risks. Identifying locations with elevated contamination risk can help target piped-water investments and interim safety measures, but collecting sufficient water quality data is costly. This study develops and validates a method to rank contamination risk across Indonesian cities using secondary data alone. Contamination probability was modelled using logistic regression applied to water quality data from 619 households in Indonesia’s 2020 national water quality survey. A composite city-level risk score was constructed by combining predicted contamination probability with the proportion of households relying on self-supplied groundwater, derived from Indonesia’s 2022 national socioeconomic survey, enabling risk ranking across all 99 cities. Dug-wells had 2.9 times greater odds of *Escherichia coli* >100 CFU/100 mL than boreholes (*p* < 0.001), and contamination risk increased significantly with population density (*p* = 0.006). Aquifer lithology and rainfall were additional predictors (*p* < 0.1). Model discrimination was acceptable (AUC 0.715, 95% CI 0.667–0.764). Independent validation against four datasets (14 locations) revealed acceptable agreement but systematic underprediction of *E. coli* exceedance probability at four locations (root mean square error of 0.116 and mean bias of −0.067), indicating the model is more reliable for identifying lower-risk than higher-risk cities. Including groundwater depth and sanitary condition data could increase model reliability. This approach provides a cost-effective screening tool for prioritising water-safety interventions where urban self-supply is widespread and data is limited.

**Supplementary Information:**

The online version contains supplementary material available at https://doi.org/10.1007/s10661-026-15909-7.

## Introduction

Self-supply from groundwater is an important source of water for 61% of households in urban Indonesia, or approximately 95 million people, who obtain a portion of their household water from private groundwater sources. Furthermore, 24% of urban households use self-supplied groundwater as their main drinking source (BPS Statistics Indonesia, 2022). Water quality is a major concern, with an estimated 68% of urban households not having access to “safely managed drinking water”, which is defined as “an improved water source that is accessible on premises, available when needed and free from faecal and priority chemical contamination” (UNICEF, 2025). The results of a nationwide study revealed that, for urban Indonesian households, the failure to achieve “safely managed drinking water” is most commonly because the water supply is not deemed free from faecal contamination due to the detection of *Escherichia coli* (Irianto, 2021). For urban households that use self-supplied groundwater as their main drinking water source, Irianto (2021) reported *E. coli* bacteria in 69% of samples. Other research on the water quality of self-supplied groundwater that focused on specific cities supports these findings (Genter et al., 2023; Indrastuti et al., 2021). A systematic review by Gruber et al. (2014) found the presence of *E. coli* in drinking water increased the risk of diarrhoea by 54% (95% CI 1.37, 1.74) so these findings on water quality imply significant health impacts.

To improve this situation, the Indonesian government plans to provide safe piped water to urban households, but as this will take time, greater benefit will be achieved by prioritising cities where risks arising from faecal contamination of self-supplied groundwater are greatest. Available water quality data indicates that the frequency of faecal contamination in groundwater sources varies according to context (Genter et al., 2021; Irianto, 2021) and studies investigating risk factors offer an explanation: many of the factors associated with higher levels of faecal contamination, including depth to groundwater, soil type, aquifer lithology and rainfall, vary geographically (Mbae et al., 2024).

The extent of water quality testing in Indonesia does not provide enough data to identify all high-risk areas, which is a common problem in many countries, especially those with limited resources (Rose et al., 2023; WHO et al., 2022). To address this, researchers have sought to use available geographic data on the factors associated with faecal contamination of groundwater to create predictions. Two main approaches have been adopted. The first uses groundwater vulnerability indices that have been developed to calculate a generic groundwater contamination risk, such as DRASTIC^1^ (Aller et al., 1987) and GOD^2^ (Foster et al., 2002). These methods calculate a score from hydrological features such as depth to groundwater and aquifer materials and some studies augment these scores with population density. While these groundwater vulnerability indices have been shown to be correlated with chemical contamination (Singh et al., 2015), correlations have not been found with microbial contamination in the two studies we found that attempted this (Jamrah et al., 2008; Nurroh et al., 2020), possibly because microbial attenuation through the vadose zone follows a first-order decay which these indices do not adequately capture (Pang, 2009). Additionally, they do not allow for temporal factors, such as rainfall, which have been shown to be an important factor in determining faecal indicator bacteria levels (Mbae et al., 2024).

The second approach involves developing a correlation between microbial water quality measurements and matching data on the risk factors (explanatory variables) expected to impact water quality. Several researchers have used this approach, applying logistic regression to predict the probability of faecal indicator bacteria exceeding a chosen cutoff level (Hynds et al., 2012; Jang, 2022; O’Dwyer et al., 2018; Poulin et al., 2020). These models are specific to the context in which they were developed. For example, Poulin et al. (2020) obtained data for Bangladesh and Uganda and found different risk factors and models for each, so a context-specific model is needed for urban Indonesia.

Logistic regression models require some conceptual understanding of likely risk factors and researchers have used several methods to identify these potential risk factors. Some researchers use a structured approach, such as the directed acyclic graph developed by Poulin et al. (2020), while others have identified potential risk factors from literature without presenting a conceptual framework for selection of risk factors (Hynds et al., 2012; Jang, 2022). A potential conceptual approach is the source-pathway-receptor (SPR) framework which was developed as part of contaminated land risk assessment practices (Vik et al., 2001) and then adopted for understanding groundwater contamination from on-site sanitation (Lawrence et al., 2001). The framework is based on an understanding that contamination arises from a source, then moves along defined pathways to a receptor. This framework provides a theory for identifying and grouping risk factors into those which are sources, those that influence transport of microbes along pathways and those factors relating to the nature of the receptor. Mbae et al. (2024) used the SPR framework to structure their systematic review of the evidence for groundwater contamination from on-site sanitation and Kelly et al. (2021) used a similar framework to fit sanitary inspection results to *E. coli* results from handpumps in Africa, but to our knowledge, the framework has not been applied to predict microbial groundwater risk.

A recent innovation in understanding the risk factors affecting microbial contamination is application of machine learning methods, which can capture complex, non-linear relationships between risk factors and contamination outcomes without the need for a conceptual framework. For example, Wu et al. (2025) applied three machine learning algorithms to a large tubewell dataset (*n* = 1495) in Bangladesh to identify and rank risk factors by significance. Such approaches show considerable promise but involve trade-offs: they typically require larger sample sizes to avoid overfitting, and their outputs are less readily interpretable than those of regression-based approaches, which produce odds ratios and confidence intervals that translate more directly into actionable risk communication. Where sample sizes are more modest and model interpretability is a priority, as is the case in the present study, logistic regression remains appropriate, offering a transparent and well-validated framework for identifying the determinants of faecal contamination risk. Both machine learning and logistic regression approaches have benefited from the increasing availability of satellite-derived geospatial datasets, which provide consistent, spatially extensive information on risk factors without reliance on ground-based surveys (Poulin et al., 2020; Wu et al., 2025) and which are particularly valuable in settings like urban Indonesia where systematic field survey data are limited or inconsistently available across cities.

The probability of a groundwater source having faecal contamination is only one component of risk, although it is the only component considered in most studies. In the engineering and science fields, risk is typically considered to be a combination of consequence and uncertainty (Aven, 2012). A more complete risk comparison for a geographic area such as a city will include both terms. Combining an assessment of uncertainty (probability of self-supplied groundwater having faecal contamination) with consequences (numbers of people using self-supplied groundwater) will provide a more complete assessment of risk that can be used to rank cities.

Research aimed at predicting the probability of faecal contamination has focused largely on rural areas, and no predictive model for cities has been developed. Jang (2022) included some urban areas in the predominantly rural catchment studied and reported that urban land use was a risk factor associated with microbial contamination; however, he did not attempt to develop different models for urban and rural areas. Because urban areas have higher population density, greater proportions of impervious land areas and fewer livestock, predictive models based on rural areas or mainly rural areas may not be applicable.

This study develops a method for using existing data on risk factors to estimate and compare the risk of faecal contamination in self-supplied groundwater across Indonesian cities. It makes three contributions to the literature. First, it applies the source-pathway-receptor framework as a principled basis for identifying and selecting risk factors, extending its use beyond evidence synthesis (Mbae et al., 2024) into predictive modelling for the first time. Second, it develops a binary logistic regression model specifically for an urban context, addressing a gap in the literature where predictive models have been developed primarily for rural or mixed settings. Finally, it moves beyond the probability of contamination at an individual source to construct a composite city-level risk score by combining predicted contamination probability with data on the proportion of the population relying on self-supplied groundwater. Together, these contributions offer a transparent method for risk assessment in low- to middle-income urban settings where self-supplied groundwater is common.

## Materials and methods

### Risk consequences

To create a faecal contamination risk rank of Indonesian cities,^3^ existing datasets were examined to quantify the two components of risk: uncertainty and consequences. For the consequences component, i.e. the size of the population potentially affected, data on the proportion of households in each city relying on self-supplied groundwater from dug-wells or boreholes were obtained from Indonesia’s National Socioeconomic Survey (BPS Statistics Indonesia, 2022). This survey used a stratified, multi-stage cluster sampling design in which primary sampling units (census blocks) are selected within regency/city strata, yielding a nationally representative sample of approximately 330,000 households across all 514 regencies and cities. In this survey, a dug-well is a groundwater source constructed by digging; it often has lined walls below ground and may have a cover (and thus categorised as a “protected dug-well”) or be open (“unprotected dug-well”). Protected and unprotected dug-wells were combined for this study due to uncertainty around the accuracy of the protection categorisation. Dug-wells may use either electric pumps or hand operated devices such as buckets for water extraction. Drilled-bores are constructed by drilling and within cities almost always use an electric pump and are covered.

### Risk uncertainty—logistic regression

**Fig. 1:**
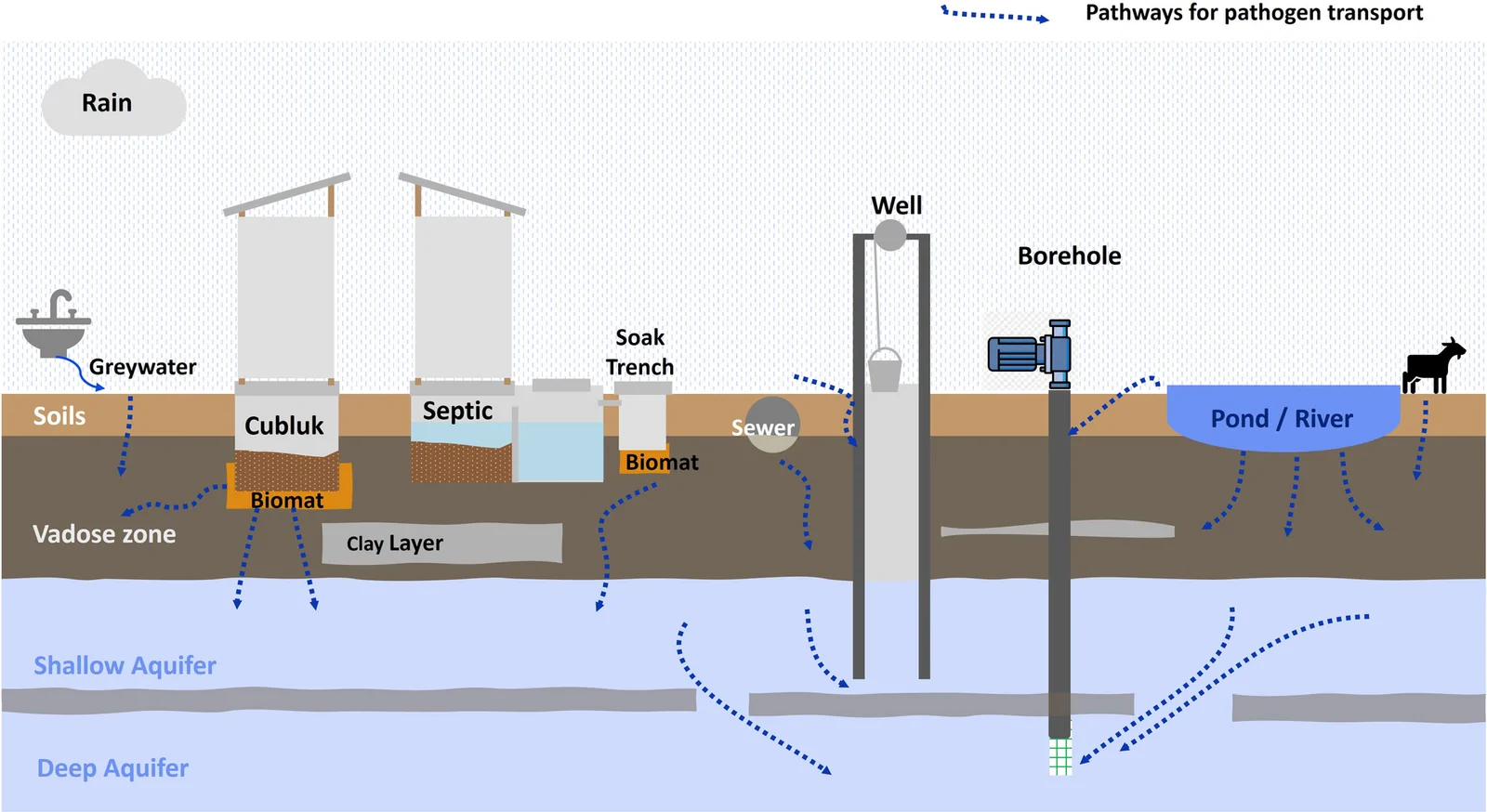
Conceptual diagram

For uncertainty, the second component of risk, the approach adopted by previous researchers (Hynds et al., 2012; Jang, 2022; O’Dwyer et al., 2018; Poulin et al., 2020) was followed, in which a statistical model is used to predict the probability that faecal indicator bacteria exceed a defined threshold (dependent variable) from existing data on risk factors (the explanatory variables). Potential risk factors affecting microbial contamination in household groundwater sources in urban areas were identified using the “source-pathway-receptor” framework to develop a conceptual model, informed by a review of academic literature (Mbae et al., 2024). Figure 1 illustrates the conceptual model.

Based on this framework, the following sources, pathways and receptors are expected to contribute to groundwater contamination in Indonesian cities:

- Sources:
  - On-site sanitation systems (90% of urban households in Indonesia use on-site sanitation (UNICEF, 2025)
  - Sewer leakage (1% of urban households in Indonesia have a piped sewer connection (UNICEF, 2025)
  - Livestock/birds
  - Untreated greywater discharge from premises (in urban Indonesia commonly only toilet waste is connected to an on-site sanitation system with greywater discharged to street drains)
- Pathways:
  - Aquifer pathway: contaminants travel through an unsaturated zone (except where a source discharges below the groundwater table) into an aquifer and then flow towards the water source (receptor). Multiple aquifers separated by aquitards, which are common in Indonesian cities, can complicate this pathway. Indonesia has one of the most diverse geological formations in the world making generalising difficult (IGRAC, 2025). Nonetheless, many cities have a shallow aquifer of less than 10 m depth and hence dug-wells are commonly used, while deeper aquifers up to 50 m deep are commonly accessed by boreholes.
  - Localised pathways: contaminants enter the receptor directly without flowing through the aquifer, for example, surface runoff entering through cracks in a dug-well wall.
- Receptors:
  - Boreholes
  - Dug-wells

Pathways and receptors provide barriers that reduce pathogen transmission with varying effectiveness. Rainfall functions as an enabling factor by increasing water flow into aquifers and receptors, potentially enhancing pathogen movement along these pathways. Indonesia is in the tropics and straddles the equator, so most of the country has a dry and wet season, though equatorial zones have year-round rainfall. Annual rainfall for cities ranges from around 1000 to 4000 mm/year.

Using this conceptual model, data available across all Indonesian cities were examined to identify variables that directly measured sources, pathways, receptors or their effectiveness as pathogen barriers. Where direct measurements were unavailable, proxy indicators were sought. Table 1 summarises the secondary data that was found for each risk factor including the source of the data and whether it directly measures the risk factor or acts as a proxy.

**Table 1:** Description of secondary data obtained for testing in regression model

Risk factor	Potential data to quantify risk factor	Source of data	Comments on data
**Sources**			
Load	Population density (people/pixel)	(Joint Research Centre & Center for International Earth Science Information Network, 2021)	Proxy for faecal load
	% land area built-up	(Joint Research Centre & Center for International Earth Science Information Network, 2021)	Proxy for faecal load
	% households in city with no sanitation	(BPS Statistics Indonesia, 2022)	Proxy for load as it captures households reported not having any facility for toilet waste, so likely direct discharge to drains, local waterways
**Pathways**			
Aquifer pathway	Land slope	Calculated from (NASA, 2020) 1 arc second global	Direct measure—greater slope increases runoff
	Depth to groundwater	(Fan et al., 2013)	Direct measure
	Aquifer lithology	Provided by Pusat Air Tanah dan Geologi Tata Lingkungan (PAG)	Direct measure. Data had four categories which were grouped into two for analysis: (solid rock + volcanic rock) and (unconsolidated sediment + limestone)
	Sanitation facility within 15 m	(Irianto, 2021)	Reflects likely shorter pathway from source to receptor
Localised pathways	Sanitary inspection score (SIS)	Derived from (Irianto, 2021)	Proxy for presence of localised pathways. Calculated by summing the number of “yes” answers in a sanitary inspection survey
Enabling impact of rainfall	Rainfall over October to November 2020	(Fick & Hijmans, 2017)	Direct measure
**Receptor**			
Type	Drinking water source type (dug-well or drilled-bore)	(Irianto, 2021)	Direct measure. Protected and unprotected dug-wells were grouped together

Among the factors identified in the conceptual model, no relevant data were found for livestock density; however, livestock in urban areas are unlikely to be a major pathogen source. Data was also unavailable on the composition of ground material within the first few metres of the unsaturated zone. No data was available on the extent of sewer leakage or overflow. Since piped sewerage serves only 1% of urban Indonesian households (UNICEF, 2025), this data gap is unlikely to influence the results.

To provide faecal indicator bacteria data suitable for statistical modelling, the household drinking water quality study (SKAM-RT), a nationwide household survey on water and sanitation conducted in 2020 as a purposively designed sub-survey of SUSENAS, was used (Irianto, 2021). SKAM-RT employed a stratified cluster sampling design inherited from the SUSENAS framework, in which SUSENAS-sampled households were revisited for water quality testing, preserving the spatial stratification across provinces and regencies that ensures national representativeness. The survey collected and analysed water samples for multiple parameters, including *E. coli*, from the primary drinking water source of each participant household. The target sample size of 21,829 households was determined by the SUSENAS sub-sampling allocation, designed to maintain representativeness at the provincial level. Of these, 847 households located within cities used either dug-wells or boreholes as their main drinking water source and therefore had data on *E. coli* which could be used in this analysis.

Spatial coordinates of households were required to match water quality observations with risk factors such as population density and geological conditions. Because some SKAM-RT household locations were unavailable, the usable sample size was reduced to 619 households. A Mann–Whitney *U* test (*p* = 0.127) showed no significant difference in *E. coli* distributions between households with known versus unavailable locations, indicating no location-based bias resulting from the exclusion of households with uncertain positions.

Previous studies have shown binary logistic regression to be an appropriate statistical approach for relating faecal indicator bacterial data to explanatory variables (Irianti et al., 2024). SKAM-RT reports *E. coli* using WHO risk categories; the high-risk threshold of >100 CFU/100 mL was selected as the binary cutoff (Bain et al., 2021), and exploratory analyses indicated that this threshold distinguished spatial differences more effectively than “detected vs. not detected”. Binary logistic regression fits data to Eq. 1.

1 $$\mathrm{l}\mathrm{o}\mathrm{g}\mathrm{i}\mathrm{t}\left(P\right)=\mathrm{l}\mathrm{n}\left(\frac{P}{1-P}\right)={\beta}_{0}+{\beta}_{1}{X}_{1}+{\beta}_{2}{X}_{2}+\dots +{\beta}_{n}{X}_{n}$$

where:

- *P* = probability of *E. coli* being >100 CFU/100 mL
- *β*_0_ = intercept
- *β*_1_ = coefficient for risk factor 1
- *X*_1_ = value of risk factor 1

For each explanatory variable, the odds ratio $(\mathrm{O}\mathrm{R})={e}^{\beta i}$ was calculated to provide an easier to understand measure of impact.

The SKAM-RT employed cluster sampling, with groups of ten adjacent households surveyed in each *desa* (urban village), yielding 192 clusters for analysis. Because groundwater contamination may be spatially correlated among nearby households sharing similar subsurface conditions, the statistical analysis required methods that account for within-cluster correlation. A generalised estimating equation (GEE) framework was therefore applied to model clustered observations (Hubbard et al., 2010), with a binary outcome indicating whether *E. coli* exceeded 100 CFU/100 mL, using a binomial family with logit link. An exchangeable working correlation structure was specified, reflecting the assumption that correlation between any two households within the same *desa* is approximately constant regardless of their relative positions. Robust sandwich standard errors were used, ensuring that regression parameter estimates remain valid even if the working correlation structure is misspecified. The scale parameter was fixed at 1, as is standard for binomial GEE models. All GEE models were fitted in SPSS version 28.0.1.1 (IBM, 2021). The events-per-variable ratio (EPV) was 32.8 (131 households exceeding the *E. coli* threshold across 4 predictor variables), satisfying recommended adequacy thresholds for logistic regression model stability (Peduzzi et al., 1996).

**Fig. 2:**
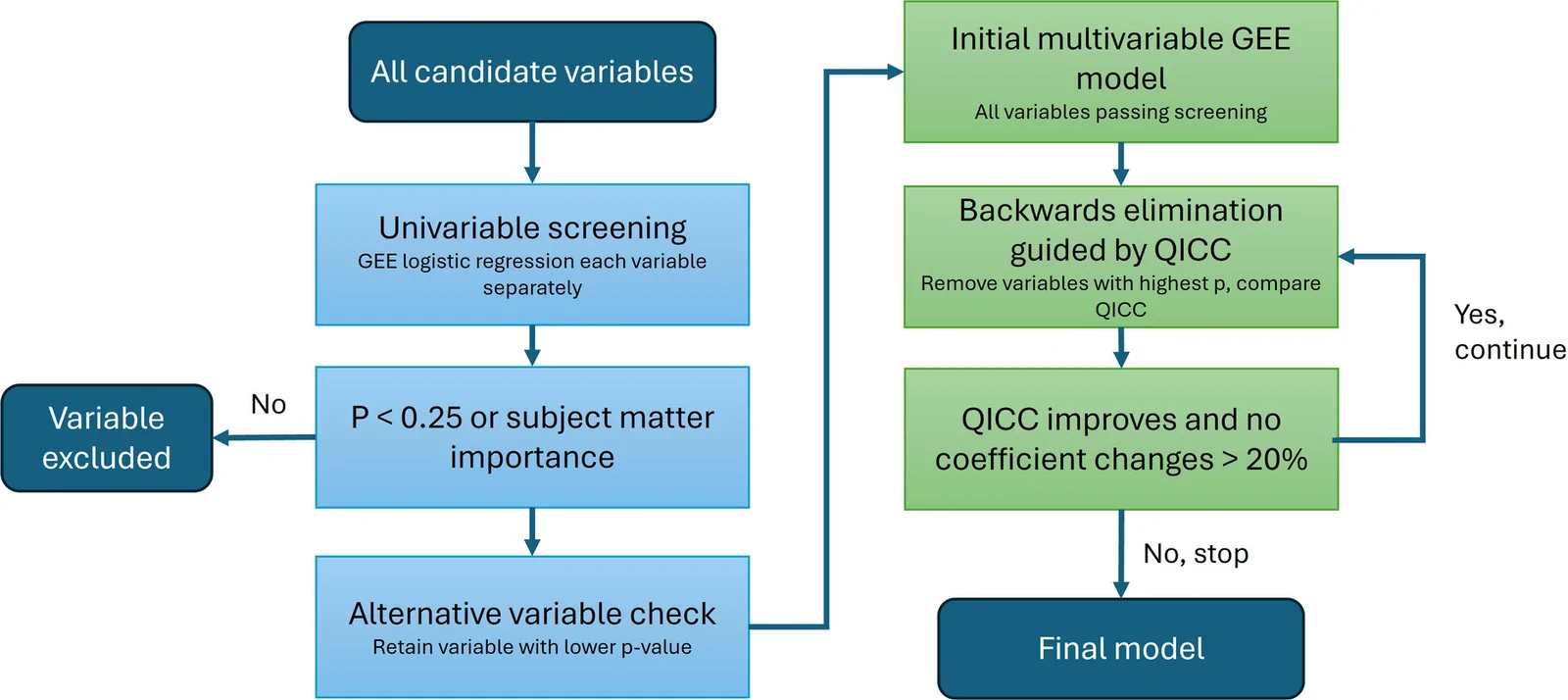
Variable selection flow diagram

The purposeful selection method (Hosmer et al., 2013) was used to identify the subset of risk factors from Table 1 for the final model. This method integrates statistical significance with subject-matter knowledge, allowing inclusion of variables with theoretical importance even when statistical evidence is marginal and the selection process is shown in Fig. 2. Aquifer lithology was significant at only *p* = 0.1 but was retained on the basis that it acts as a spatial proxy for locations likely to have shallow groundwater. One assessment of this correspondence is that 76% of the locations having the aquifer lithology type associated with highest *E. coli* (unconsolidated sediment + limestone) have an elevation under 100 m, while only 31% of locations with solid or volcanic rock have an elevation under 100 m.

A geographic information system (QGIS Development Team, 2024) was used to extract values for risk factors such as population density, topography and lithology at the locations of the water quality measurements. The corrected quasi likelihood under independence model criterion (QICC) calculated by SPSS software was used to guide the decision on the final model with the best fit. For rainfall, the selection of time-period was constrained by available data as only the sampling month, not the exact date, was available for each household.

Multicollinearity among the four chosen predictor variables was assessed using the Variance Inflation Factor (VIF), calculated from an auxiliary ordinary least squares regression on the same predictors. All VIF values were below 1.1 (population density = 1.06; rainfall = 1.03; receptor type = 1.05; aquifer lithology = 1.02), indicating negligible multicollinearity (threshold VIF < 5) (Hair, 2019). The linearity of the log-odds assumption for continuous predictors was assessed using the Box-Tidwell test (Box & Tidwell, 1962), in which interaction terms of the form *X* * ln(*X*) were computed for population density and rainfall and added to the GEE model. Neither interaction term was statistically significant (population density: *p* = 0.233; rainfall: *p* = 0.221), indicating that the linearity assumption was satisfied for both predictors.

### Sensitivity analysis for variables excluded due to data limitations

Three variables identified in the conceptual model as potentially important predictors could not be included in the final model due to data limitations which are summarised in Table 2, along with the reason for exclusion and the expected direction of bias on model predictions.

**Table 2:** Excluded variable bias summary

Risk factor	Reason for exclusion	Expected bias and magnitude
Depth to groundwater	Only available dataset (Fan et al., 2013) showed no correspondence with field measurements (*r* = −0.01, *p* = 0.921)	Upward bias (underprediction) in cities with shallow water tables (e.g. flood-prone coastal cities); magnitude unquantifiable
Sanitary inspection score (SIS)	Household-level data unavailable at city scale for prediction	Upward bias; scenario analysis indicates absolute probability shift of 0.04–0.09 across likely SIS range (Supplementary Table S1)
Vadose zone properties	No data available at any applicable spatial scale	Direction and magnitude uncertain; influence may be reduced in urban settings where ground disturbance disrupts natural soil properties (Invik et al., 2019; O’Dwyer et al., 2018)

To quantify the potential impact of the omitted sanitary inspection score (SIS), a supplementary GEE model was fitted including SIS alongside the four main predictors on the 573 households for which SIS data were available. Its inclusion produced negligible change in the odds ratios of the four main predictors (e.g. well/bore type changed from 2.90 to 2.69), confirming that SIS acts as an independent contributor rather than a confounder. A scenario analysis applying both model equations to covariate profiles at the 25th, 50th and 75th percentiles of city-level predictor values, with SIS varied from 0 to 4 (the 90th percentile of observed household values), showed predicted contamination probability increased from 0.09 to 0.15 at the median city profile, an absolute shift of 0.06; the equivalent shift at higher-risk profiles reached 0.09 (Supplementary Table S1).

**Fig. 3:**
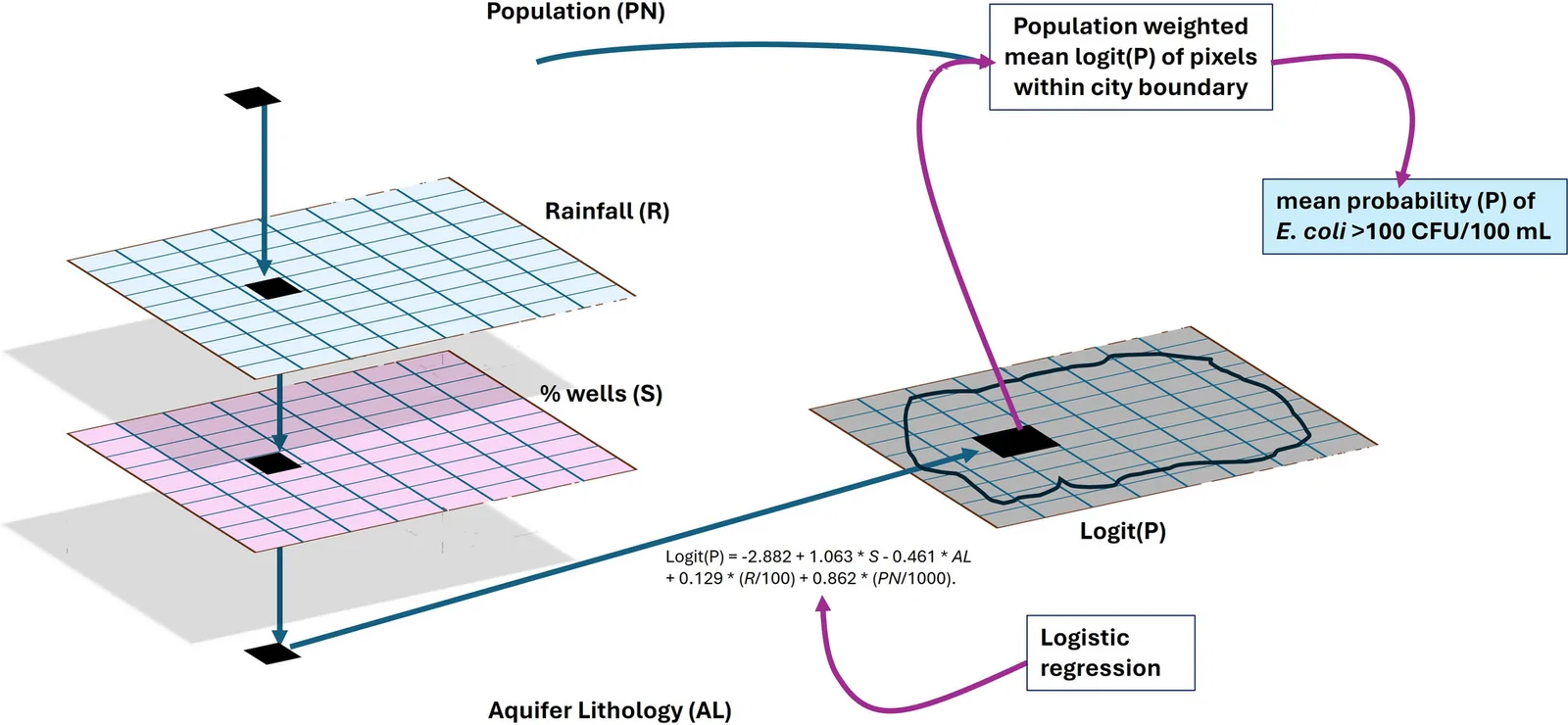
Flowchart for calculating mean probability

After the logistic regression model had been fitted, the following steps were used to estimate the average probability of *E. coli* >100 CFU/100 mL for each city (illustrated in Fig. 3):

- GIS raster layers were created for each explanatory variable included in the logistic regression model, with each raster pixel assigned the spatially corresponding variable value.
- The fitted model was applied to each pixel to calculate logit(*P*). The pixel size (280 m × 315 m) corresponded to the resolution of the underlying risk factor data.
- A mean logit(*P*) was computed for each city administrative boundary. Because many city boundaries include sparsely populated areas, a population-weighted mean was calculated instead of a simple area average.
- The population-weighted mean logit(*P*) was transformed to obtain the mean probability (*P*) of *E. coli* >100 CFU/100 mL for each city.

### Risk ranking

Two approaches were utilised to develop a risk ranking. The first approach plotted the two components of risk—uncertainty and consequence—on a two-dimensional scatterplot. The second approach calculated a risk score as the product of the city-level predicted contamination probability and the proportion of households using self-supplied groundwater for drinking, which was adopted to provide a single score for comparisons and reflects a measure of the proportion of households in a city likely to have *E. coli* >100 CFU/100 mL in source water for drinking. This formulation assumes the two components are unrelated at the city level; Pearson and Spearman correlations between predicted contamination probability and self-supply proportion across cities, reported in the Results, showed no significant association, supporting this assumption.

Risk tier boundaries were defined at scores of 0.05 and 0.01, thresholds chosen to reflect practical rather than statistical criteria, as the continuous distribution of risk scores did not exhibit natural breaks. Uncertainty in risk scores was quantified by combining 95% confidence intervals on predicted contamination probability, derived from propagation of the GEE coefficient covariance matrix through the logit equation, with 95% binomial confidence intervals on groundwater usage proportions derived from SUSENAS sample sizes. Since the two components are derived from independent surveys, their uncertainties are uncorrelated. However, these intervals capture only GEE coefficient uncertainty and survey sampling uncertainty; they do not incorporate omitted-variable bias, seasonal mismatch between the calibration period and any future application, or model-structure uncertainty arising from modelling assumptions such as the choice of functional form, predictor set and spatial aggregation method. The reported intervals should therefore be interpreted as lower bounds on total prediction uncertainty.

### Validation of the uncertainty component of risk

Validation of the uncertainty term, the predicted average probability of *E. coli* >100 CFU/100 mL, was undertaken using four datasets independent of the calibration data.

- Jakarta Environmental Protection Agency dataset: routine monitoring data from the five municipalities of Jakarta, consisting of 267 sampling points collected between 2017 and 2021. Boreholes accounted for 95% of sampling points, with the remainder dug-wells. As with SKAM-RT, sampling points represented community-used water sources rather than purpose-built monitoring wells.
- Metro City and Bekasi dataset: a dataset collected in 2020–2021 consisting of 561 samples from Metro City and 568 samples from Bekasi, obtained from previous research by some of the authors (Genter et al., 2022).
- Yogyakarta environmental monitoring dataset: a 2021 dataset from the Yogyakarta environmental monitoring report (Yogyakarta, 2022).
- Additional Yogyakarta dataset: one location from Yogyakarta reported by Sriyono et al. (2019).

For each dataset, a “measured” proportion of samples with *E. coli* >100 CFU/100 mL was calculated using the same method applied to the SKAM-RT dataset. A corresponding modelled value was generated using the values of the explanatory variables for the same geographic area along with the actual proportion of dug-wells present in each dataset. Prediction error bounds were calculated using the standard error formula:

2 $$\mathrm{S}\mathrm{E}=\surd ({x}_{j}*V{x}_{j})$$

where *x*_*j*_ is the vector of explanatory variables for observation *j* and *V* is the covariance matrix of the model coefficients.

## Results

Table 3 presents descriptive statistics for the 619 households in the analytical sample, stratified by *E. coli* outcome. The overall prevalence of *E. coli* >100 CFU/100 mL was 21.2%. Households with *E. coli* exceedance had higher median population density and cumulative October/November rainfall, and a greater proportion used dug-wells, compared with uncontaminated households.

**Table 3:** Descriptive statistics of the analytical sample (*n* = 619), stratified by *E. coli* outcome

Variable	Overall (*n* = 619)	*E. coli* ≤100 CFU/100 mL (*n* = 488, 78.8%)	*E. coli* >100 CFU/100 mL (*n* = 131, 21.2%)
**Continuous variables—mean (SD); median [IQR]**			
Population density (households/km^2^)*	641 (468); 586 [202–984]	610 (448); 547 [192–959]	759 (521); 764 [335–1135]
Cumulative rainfall, Oct–Nov (mm)*	548 (241); 536 [386–698]	531 (236); 507 [381–683]	614 (248); 642 [422–764]
Land slope (degrees)	1.1 (1.5); 0.4 [0.3–1.0]	1.1 (1.6); 0.4 [0.3–1.1]	0.9 (1.0); 0.5 [0.3–0.9]
Depth to groundwater (m)	3.0 (5.8); 1.1 [0.3–2.6]	3.2 (6.2); 1.0 [0.3–2.5]	2.3 (4.0); 1.1 [0.4–2.8]
Sanitary inspection score (SIS)‡ (*n* = 573)	1.6 (1.6); 1.0 [1.0–2.0]	1.5 (1.5); 1.0 [1.0–2.0]	2.1 (1.8); 2.0 [1.0–3.0]
**Categorical variables**—*n* (%)			
Well type*			
Drilled-bore	358 (57.8%)	307 (62.9%)	51 (38.9%)
Dug-well	261 (42.2%)	181 (37.1%)	80 (61.1%)
Aquifer lithology*			
Limestone or unconsolidated sediment	225 (36.3%)	175 (35.9%)	50 (38.2%)
Volcanic or consolidated rock	394 (63.7%)	313 (64.1%)	81 (61.8%)
Sanitation facility within 15 m of source§ (*n* = 573)			
No	203 (35.4%)	165 (37.0%)	38 (29.9%)
Yes	370 (64.6%)	281 (63.0%)	89 (70.1%)

*Variable retained in final GEE model

‡§Higher score indicates more sanitary faults; excluded from final model as city-level data unavailable for prediction. The same 46 households had missing values for both SIS and sanitation facility proximity variables

*IQR*, interquartile range; *SD*, standard deviation

### Uncertainty component—logistic regression model and risk factors

Five risk factors: drinking water source type, aquifer lithology, rainfall during the 2 months preceding and overlapping sampling, population density and SIS, showed statistically significant associations (*p* < 0.1) with the likelihood of *E. coli* exceeding 100 CFU/100 mL. Table 4 summarises all assessed risk factors, presenting the odds ratios (OR) and significance values (*p*) both for the full model (including all available variables) and for the reduced model containing only the four selected risk factors (as discussed in methods, SIS was excluded despite its significant association due to lack of representative city data).

**Table 4:** Risk factor associations with *E. coli* exceedance: full model and final model compared

Risk factor	Potential data to quantify risk factor	Model that includes all risk factors		Final model—selected risk factors only	
		Adjusted odds ratio	*p*	Adjusted odds ratio (95% CI)	*p*
Sources					
Load	Population density (people/pixel)	2.17	0.017	2.37 (1.3–4.4)	0.006
	Land area built-up (%)	1.01^a^	0.16	–	–
	Households in city without sanitation (%)	3.1	0.41	–	–
Pathways					
Aquifer pathway	Land slope (%)	0.97	0.80	–	–
	Depth to groundwater (m)	1.04	0.14	–	–
	Aquifer lithology (category)	0.64	0.12	0.63 (0.4–1.1)	0.10
	Sanitation facility within 15 m (yes/no)	0.77	0.34	–	–
Localised pathways	Sanitary inspection score (SIS) (integer)	1.18	0.009	–	–
Enabling impact of rainfall	Cumulative rainfall for Oct/Nov 2020 (mm)	1.10	0.21	1.14 (1.0–1.3)	0.049
Receptor					
Type	Drinking water source type (dug-well or drilled-bore)	2.81	<0.001	2.90 (1.8 to 4.8)	<0.001

^a^The % land built-up and population density were not used together in the same modelling run but were swapped to determine which had a more significant association with *E. coli*

The best fit model was:

3 $$\mathrm{Logit}\left(P\right)=\mathrm{-2.882}+\mathrm{1.063}*{S}\mathrm{-0.461}*{AL}+\mathrm{0.129}*(R/100)+\mathrm{0.862}*({PN}/1000)$$

where:

- *S* = whether the drinking water source is a dug-well (1) or a drilled-bore (0)
- *AL* = aquifer lithology is solid or volcanic rock = 1 otherwise 0
- *R* = total rainfall from October to November in mm
- *PN* = population per pixel^4^

### Risk ranking

**Fig. 4:**
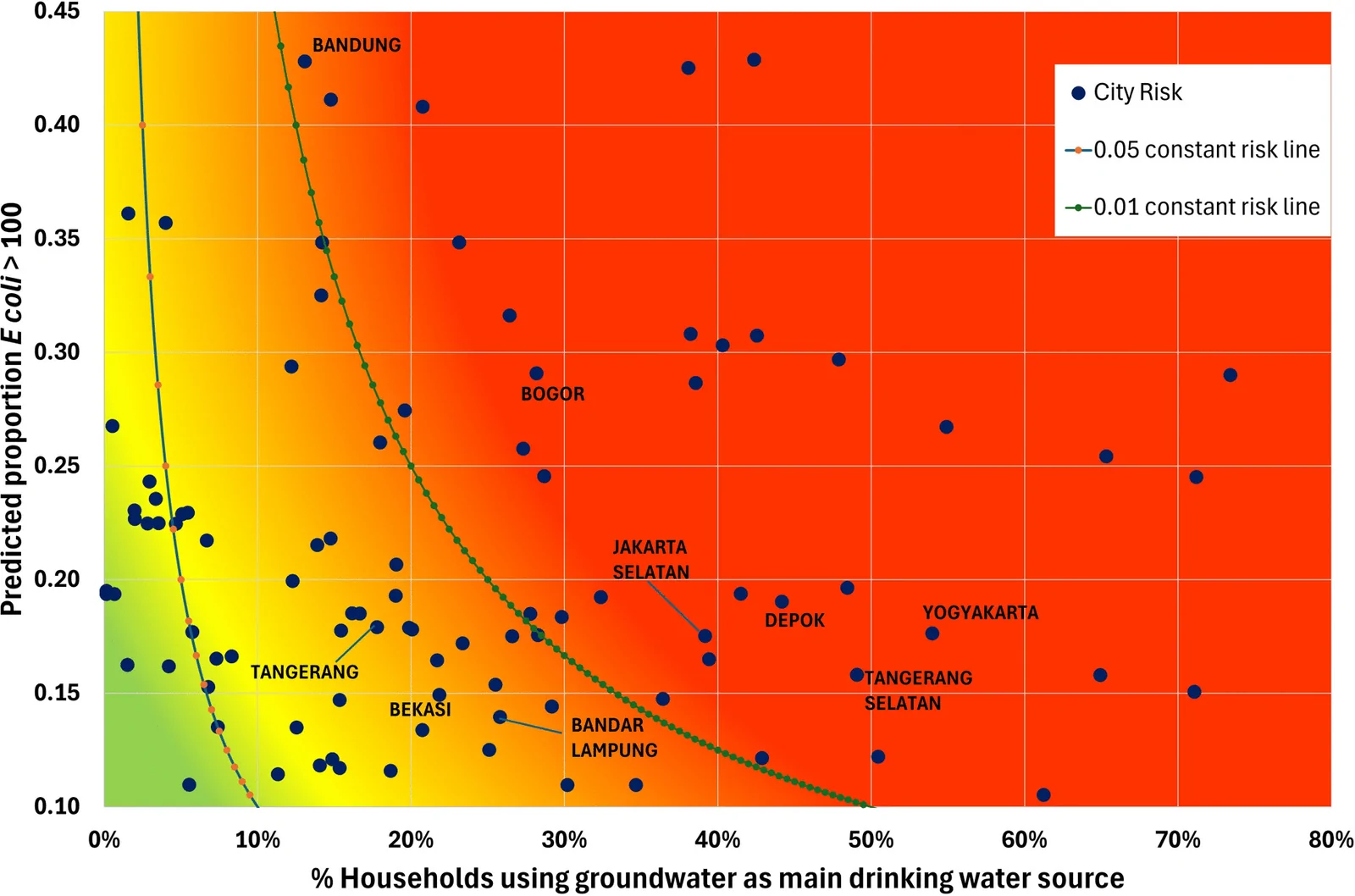
Risk ranking diagram

**Fig. 5:**
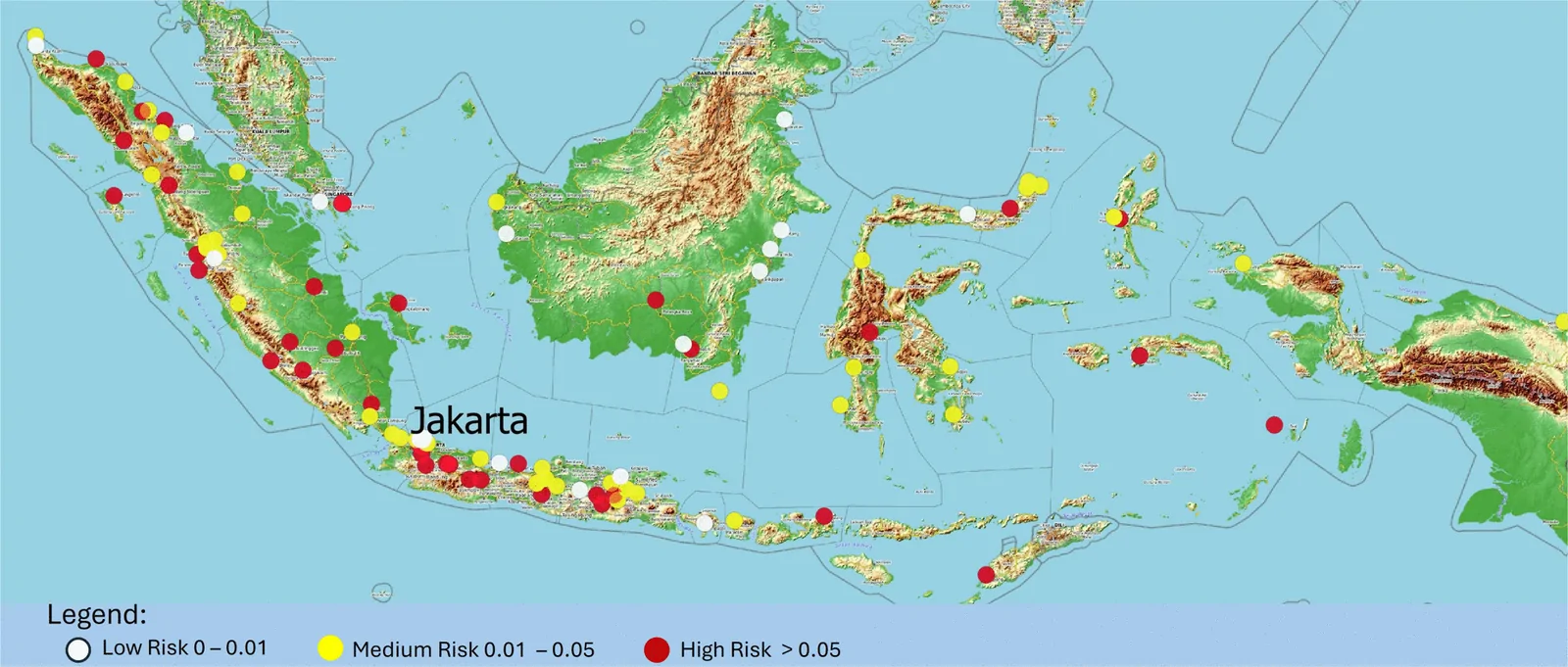
Indonesia with cities by composite risk score in three risk categories

Figure 4 shows the plot for each city of uncertainty (predicted probability of groundwater *E. coli* >100 CFU/100 mL) against the consequence (percentage of households using groundwater as their main drinking water source) while Fig. 5 shows cities grouped into three levels of risk and plotted on a map of Indonesia. The two components of the risk score were effectively independent across the 99 cities (Pearson *r* = −0.033, *p* = 0.749; Spearman rho = −0.091, *p* = 0.369), confirming the validity of the multiplicative formulation. Of 99 cities, 19 were robustly classified as high risk (lower 95% CI ≥ 0.05), 5 as robustly medium risk (95% CI between 0.01 and 0.05) and 5 as robustly low risk (upper 95% CI < 0.01), and the remaining 70 had tier classifications that were sensitive to uncertainty and should be interpreted accordingly. Full results, including confidence intervals for all cities, are provided in Supplementary Table S2.

### Calibration and validation

The area under the ROC curve (AUC) was 0.715 (95% CI 0.667–0.764), indicating acceptable discrimination between households with and without *E. coli* exceedance (Hosmer et al., 2013). Model calibration was assessed using a calibration plot of mean predicted probability against observed proportion across deciles of predicted risk (Supplementary information, Fig. S1); most deciles fell close to the line of perfect calibration, with a tendency towards underprediction in the highest deciles.

Classification is presented in Table 5. At the Youden-optimal cutoff of 0.224, the model correctly classified 430 of 619 households (overall accuracy 69.5%), with a sensitivity of 0.618, specificity of 0.715, positive predictive value (PPV) of 0.368 and negative predictive value (NPV) of 0.875. The relatively low PPV reflects the low prevalence of the outcome (21%) rather than poor model performance; the high NPV indicates the model is more reliable for identifying lower-risk areas than for confirming high-risk ones.

**Table 5:** Classification table

	Predicted < threshold	Predicted ≥ threshold
Observed *E. coli* ≤100 (*n* = 488)	349 (true negative)	139 (false positive)
Observed *E. coli* >100 (*n* = 131)	50 (false negative)	81 (true positive)

**Fig. 6:**
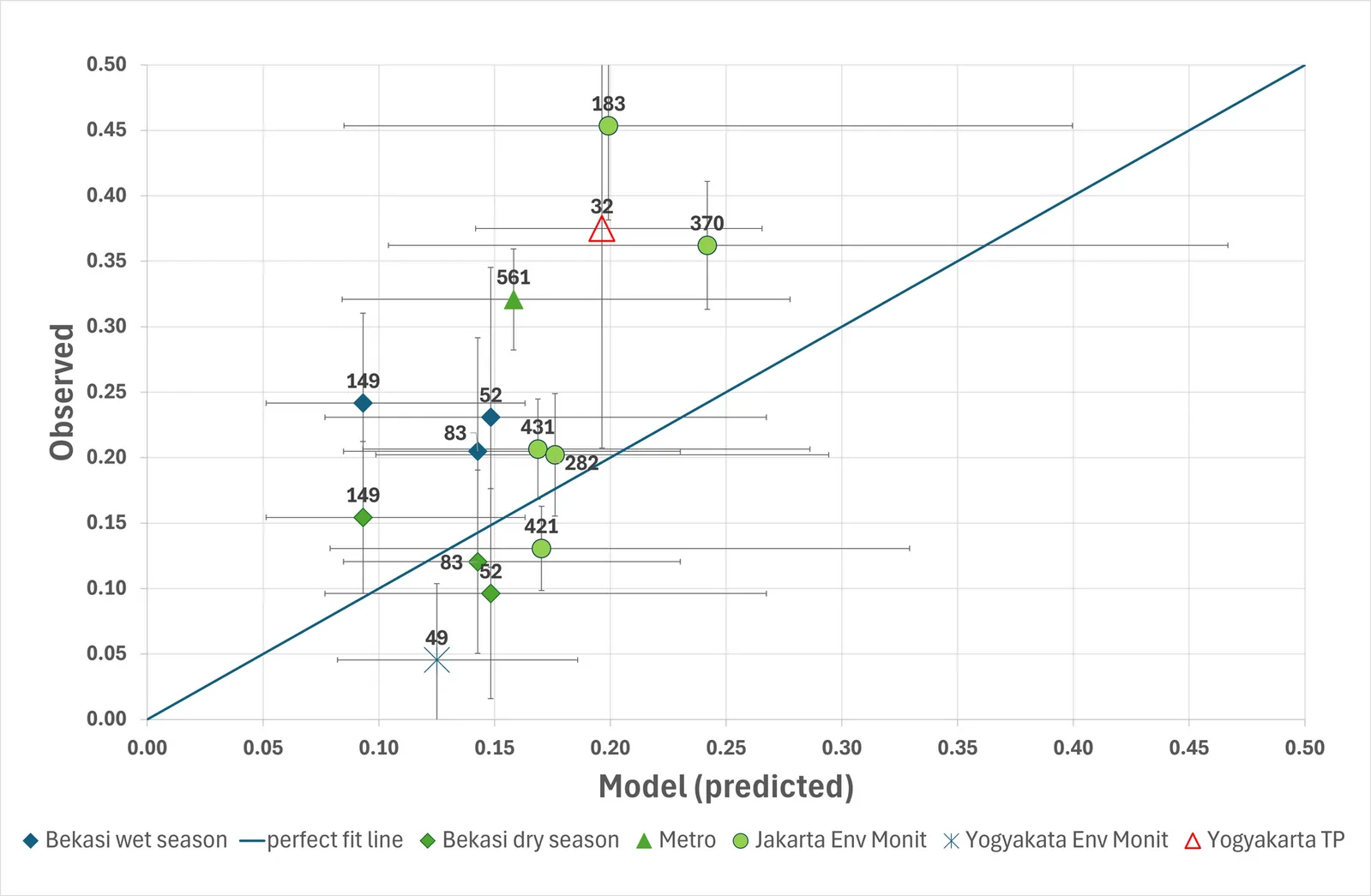
Comparison of the model results and results from other datasets (numbers next to points are sample size. Vertical bars are sampling uncertainty (√(*p*(1 − *p*)/*n*)) and horizontal bars are model parameter uncertainty)

Figure 6 compares the model-predicted proportion of samples with *E. coli* >100 CFU/100 mL against proportions observed in cities from the four independent datasets. Each point includes horizontal error bars representing model parameter uncertainty and vertical error bars representing sampling uncertainty (±1.96 × SE, where SE = √(*p*(1 − *p*)/*n*)). For 10 of 14 validation locations, the observed proportion fell within the model confidence interval, indicating acceptable agreement. At four locations: Metro, Bekasi cluster 3 (wet season), Jakarta Utara and Yogyakarta Tegal Panggung, the observed proportion exceeded the model upper confidence limit, indicating systematic underprediction. For the Yogyakarta environmental monitoring dataset, the measured proportion was lower than the modelled value, though the confidence intervals overlapped.

Quantitative error metrics across all 14 validation locations were mean bias −0.067 (model underpredicts by 6.7 percentage points on average), mean absolute error 0.095 and root mean square error 0.116. The bias was not uniformly distributed—underprediction was most pronounced at locations with the highest measured contamination proportions, consistent with the role of missing risk factors discussed in the following section.

## Discussion

### Risk factors—sources

Among the source-related risk factors, population density showed a statistically significant association with *E. coli* contamination (*p* = 0.006), with higher population density corresponding to a greater probability of *E. coli* >100 CFU/100 mL. This finding is consistent with Poulin et al. (2020) for Uganda but contrasts with their results for Bangladesh. In Bangladesh, Poulin et al. (2020) used population density averaged over both 500 m and 2 km radii around water sources but found no association and suggested this may be due to contamination being attenuated more rapidly in the ground conditions of Bangladesh. The spatial scale used in the present study (approximately 280 m) aligns with that interpretation. Jang (2022) did not find a correlation with population density but did find urban land use to be a significant predictor for their high cutoff case (*E. coli* > 248 CFU/100 mL). Since the current analysis focused exclusively on urban settings, where livestock and other rural contamination sources are minimal, population density may be a more reliable indicator of contaminant loading.

Among the source-related variables excluded from the final model, the percentage of built-up land showed a less significant association than population density, and the two variables were not included together due to lack of independence. The proportion of households without sanitation could plausibly indicate greater *E. coli* loading from untreated waste discharged to the environment, yet no significant association was observed. It is possible that in some contexts lack of formal household sanitation, while resulting in more contamination of surface waters, may keep faecal contamination away from groundwater when compared to on-site sanitation that discharges waste with limited treatment below the surface.

### Risk factors and pathways

Among the pathway-related risk factors, only aquifer lithology and rainfall were retained in the final model. Although both were significant at the 90% confidence level rather than the more conventional 95%, they were included because their importance is strongly supported by theory and previous research. The dataset classified aquifer materials into four categories that in this study were consolidated into two groups: (1) solid and volcanic rock and (2) unconsolidated sediment and limestone. Trial models using alternative groupings showed minimal differences in odds ratios between solid and volcanic rock, while the limestone category contained only 16 data points. Locations underlain by solid or volcanic rock exhibited 40% lower odds of *E. coli* >100 CFU/100 mL than locations underlain by unconsolidated sediment or limestone. This contrasts with O’Dwyer et al. (2018), who reported lower *E. coli* detection odds in sand/gravel than in bedrock, and with broader literature indicating greater bacterial removal in finer sediments than in fractured rock (Mbae et al., 2024). One possible explanation is that the broad categories used to describe aquifer lithology inadequately capture material-specific removal processes. However, a more likely explanation supported by the cross tabulation showing unconsolidated sediment/limestone more frequently occurs in elevation under 100 m is that unconsolidated sediments and limestone correspond primarily to low-lying coastal areas and river valleys, where shallow groundwater is common, a factor strongly associated with elevated contamination (Mbae et al., 2024) so the association may therefore reflect this co-occurrence rather than any direct removal effect. To test whether the lithology association might be explained by terrain characteristics, a slope × lithology interaction term was added to the GEE model alongside slope as a main effect. Neither the interaction term (*p* = 0.879) nor the slope main effect (*p* = 0.958) was significant, and the lithology odds ratio was essentially unchanged (0.649 versus 0.631 in the final model). However, slope is an imperfect proxy for groundwater depth, and the confounding hypothesis cannot be fully evaluated without accurate national-scale depth-to-groundwater data; the only available proxy (Fan et al., 2013) showed no meaningful correspondence with field measurements and could not be used. Until accurate depth-to-groundwater data are available nationally, aquifer lithology in this model is best understood as a tentative spatial proxy for broader hydrogeological setting rather than a direct mechanistic predictor of microbial attenuation.

The inclusion of rainfall is consistent with findings from other predictive models (Hynds et al., 2012; Jang, 2022; O’Dwyer et al., 2018; Poulin et al., 2020) and from numerous associational studies (Akple et al., 2011; Cronin et al., 2006; Foster et al., 2019; Howard et al., 2003). The relevant rainfall time window is complex; previous research suggests that antecedent rainfall over periods ranging from several days to 1 month is influential (Invik et al., 2019; Murphy et al., 2020). In this analysis, rainfall data from the 2 months preceding and overlapping the sampling period was used as exact sampling dates for individual households were unavailable. Alternative timeframes such as annual and 1 month were tested, but due to a lack of data on exact sampling dates, shorter timeframes of a few days could not be tested. The strong evidence base supports the variable’s inclusion. Future analyses incorporating more precise rainfall timing would help refine its role in groundwater contamination.

The pathway risk factors not included in the model because there was no significant association, even at the 90% confidence level, were land slope, depth to groundwater and the presence of a sanitation facility within 15 m. Given that many of the sources of faecal contamination are expected to be from sanitation systems that typically have subsurface discharge, the lack of association with land slopes is consistent with the findings of Hynds et al. (2012), the only authors who looked at this risk factor. The lack of association with the presence of a household sanitation facility within 15 m of the drinking water source is consistent with mixed findings by other researchers (Mbae et al., 2024) and may be partly due to the presence of many such systems in an urban setting, with some just inside and some just outside the 15 m distance.

SIS is derived from visual inspection criteria that identify features increasing the likelihood of contamination through localised pathways, including cracks in protective structures, poor drainage and nearby pollution sources. The SIS questions largely follow WHO sanitary inspection guidelines, with minor differences because SKAM-RT preceded the release of the 2024 guidelines (WHO, 2024). SIS was significantly associated with *E. coli* exceedance in both univariable (*p* = 0.015) and multivariable analyses (OR = 1.15, 95% CI 1.02–1.30; *p* = 0.009), consistent with a systematic review by Kelly et al. (2020) in which 12 of 25 studies reported significant associations between faecal indicator bacteria and sanitary scores, and with Hynds et al. (2012) who documented a highly significant association (*p* < 0.001) between dug-well conditions and thermotolerant coliforms. SIS could not be included in the final predictive model because city-level data are unavailable; the implications of this omission for model predictions are discussed in the validation section below.

### Risk factors—receptors

The receptor being a dug-well rather than a drilled-bore was significantly associated (*p* < 0.001) with *E. coli* >100 CFU/100 mL. This finding aligns with the literature demonstrating higher contamination levels in dug-wells than in boreholes (Genter et al., 2023; O’Dwyer et al., 2018). O’Dwyer et al. (2018) found receptor type (hand dug-well versus drilled-bore) to be the explanatory variable with the highest odds ratio (8.8 compared with 2.9 in this study). Poulin et al. (2020) did not include this variable but noted that their Bangladesh data contained only tubewells (boreholes in the terminology of this paper) and acknowledged this as a possible source of error in Uganda. The potential explanations are that dug-wells typically access shallower groundwater, which has shorter pathways with contamination sources and that wells typically have more opportunities for contamination via localised pathways than boreholes as discussed below.

### Model validation

A comparison with independent data revealed that the measured results increased with the predicted results, but the model systematically underestimated contamination proportions at locations with higher observed *E. coli* exceedance rates. Quantitative validation across 14 independent locations yielded a mean bias of −0.067, mean absolute error of 0.095 and RMSE of 0.116, with statistically significant underprediction at four locations: Metro, Bekasi cluster 3 (wet season), Jakarta Utara and Yogyakarta Tegal Panggung (TP). At the Youden-optimal classification threshold of 0.224, the model achieved sensitivity of 0.618 and specificity of 0.715, with a high negative predictive value (0.875) indicating that the model is more reliable for identifying lower-risk areas than for confirming individual high-risk sites. Possible sources of the observed underprediction are missing risk factors due to insufficient data availability, water quality sampling in either the calibration or validation datasets not fully reflecting actual water quality, or limitations in the method of aggregating household-level contamination probability to the city scale. We consider these in turn in the following paragraphs.

The inability to include SIS as a measure of contamination via localised pathways is one likely explanation for underprediction at high-risk locations. SIS was a significant independent predictor of *E. coli* exceedance and a scenario analysis confirmed that omitting it introduces an upward bias of 4 to 9 percentage points in predicted contamination probability, with larger effects at higher baseline risk profiles (Supplementary Table S1), a magnitude consistent with the observed underprediction at high-risk validation sites. Three of the locations showing statistically significant underprediction all had a high proportion of dug-wells relative to bores: Metro (69%), Yogyakarta TP (47%) and Jakarta Utara (39%), all above the median across cities (32%). Previous research has shown that dug-wells with sanitary faults are specifically associated with *E. coli* contamination, whereas equivalent associations have not been found for drilled-bores (Genter et al., 2022). This makes poor dug-well condition a plausible explanation for the observed bias at these sites.

Two other risk factors that, from the conceptual model, are expected to be important but are not included in the model are depth to groundwater and type of material in the unsaturated zone. Jakarta Utara’s higher-than-expected rate of *E. coli* >100 CFU/100 mL may be due to the lack of accurate depth to groundwater in the model. Jakarta Utara has a high flood risk (Ihsan & Ilfan, 2025), and a shallow groundwater table creates conditions often associated with high levels of contamination (Mbae et al., 2024). The potential error from not including the type of material in the unsaturated zone is less certain. Other researchers (Invik et al., 2019; O’Dwyer et al., 2018) have reported significant associations between soil properties and *E. coli*, but their studies focused on rural areas. In urban areas, where extensive ground disturbance has occurred, soil properties may be less influential.

Numerous authors have observed temporal variability in faecal indicator bacteria results taken from the same water source, sometimes with discernible seasonal trends, sometimes without (Genter et al., 2023). The SKAM-RT data used to build the model included a single sample taken between November and December 2020. Some of the comparison datasets had multiple data points from the same locations over a period; some had only one sample, and none was completely aligned with the SKAM-RT sampling period. The Bekasi wet and dry season clusters illustrate this directly and it is a likely explanation for Bekasi cluster 3 (wet season) being one of the sites with significant underprediction. The same three spatial clusters show markedly different measured proportions between seasons, yet the model produces identical predictions for both, as it contains only rainfall for one period, allowing geographic variation in rainfall to be included, but not seasonal variation. This is a common challenge in environmental modelling, where perfect alignment between datasets is rarely achievable.

When analysing GIS data such as population density, typically provided at predefined spatial resolutions, the “modifiable areal unit problem” (Dark & Bram, 2007) can arise, whereby different analytical results may occur depending on the resolution used. To assess the potential influence of this issue, models were tested using population density averaged over multiple radii. Across radii ranging from approximately 280 m to 2 km, the odds ratio showed minimal variation. This indicates that the chosen pixel size of 280–315 m is appropriate and that the modifiable areal unit problem is unlikely to be a major source of bias in the results.

### Limitations

Several limitations should be considered when interpreting the results of this study. First, three potentially important predictors could not be included in the final model due to data unavailability: depth to groundwater, vadose zone properties and sanitary condition of water sources. The absence of these variables most likely explains the systematic underprediction observed at higher-risk locations, particularly those with high proportions of dug-wells. Second, the model was calibrated on a single cross-sectional dataset collected during one wet season (November–December 2020) and contains no seasonal term; its predictions therefore represent an average contamination probability rather than season-specific risk. Third, the validation datasets were not temporally aligned with the calibration data, introducing sampling uncertainty that limits the precision of validation comparisons. Fourth, aggregating household-level predicted probabilities to the city scale assumes that the SKAM-RT sample is representative of each city’s self-supplied groundwater users, which may not hold where sampling was spatially clustered within cities. Finally, the model was developed and validated exclusively for urban settings in Indonesia and should not be applied directly to rural contexts or other countries without re-calibration.

### Guidance for practitioners

These rankings are intended to support prioritisation of investigation and intervention resources rather than serve as definitive determination of water safety. The model should be seen as a screening tool that is more reliable for identifying lower-risk cities. With the low discrimination of the model, city rankings in the middle of the distribution are indistinguishable. Of the 99 cities, 19 are robustly in the high-risk category, 5 robustly in the medium-risk category and 5 robustly in the low-risk category. Hence, the map in Fig. 5 is only indicative for most cities. Cities in the high-risk tier should be prioritised for ground-truthing through targeted water quality testing and sanitary inspection, with particular attention to areas with high proportions of dug-wells. The high negative predictive value of the model (NPV = 0.875) provides reasonable confidence that lower-risk cities are unlikely to have widespread contamination, though local effects such as macropore flows resulting in elevated contamination cannot be excluded. Practitioners should note that the model operates at the city scale and cannot identify individual high-risk households; household-level risk assessment requires direct water quality testing or sanitary inspection. Rankings should be updated as improved data becomes available, particularly city-level SIS and accurate depth-to-groundwater measurements. While proxies like population density inherently simplify the source-pathway-receptor dynamics, they remain the most feasible instruments for nationwide ranking in data-scarce environments.

## Conclusions

This study developed a method for using existing secondary data on risk factors to estimate and compare the risk of faecal contamination in self-supplied groundwater across Indonesian cities, making three contributions to the literature. First, the source-pathway-receptor framework was used as a principled basis for risk factor selection in predictive modelling. Second, it developed a binary logistic regression model specifically for an urban context, addressing a gap where existing predictive models have focused primarily on rural or mixed settings. Third, it moved beyond individual-source contamination probability to construct a composite city-level risk score by combining predicted contamination probability with population exposure data.

The regression model identified four significant predictors of elevated *E. coli* risk: population density, well type, aquifer lithology and preceding and overlapping rainfall. Higher population density and dug-well use were the predictors with the most significant association, and these findings can directly inform prioritisation of piped-water investments and interim safety measures towards cities with higher population density and greater reliance on dug-wells for self-supply. The association between unconsolidated sediments and limestone aquifers and increased *E. coli* risk warrants cautious interpretation, as these lithologies correspond largely to low-lying coastal areas characterised by shallow groundwater, and further research is needed to disentangle lithological and hydrogeological effects.

The model has important limitations that must be acknowledged. Three potentially important predictors: depth to groundwater, vadose zone properties and sanitary condition of water sources, could not be included due to data unavailability, and their absence most likely explains the systematic underprediction observed at higher-risk cities. The model was calibrated on a single wet-season cross-sectional dataset and contains no seasonal term, meaning predictions represent average contamination probability rather than season-specific risk. Validation against independent data yielded acceptable overall performance (AUC 0.715, RMSE 0.116) but statistically significant underprediction at four locations with high proportions of dug-wells, reinforcing that the rankings should be treated as a screening assessment and relative prioritisation tool rather than a definitive assessment of water safety. The model is more reliable for identifying low-risk cities than high-risk cities. The model produces a citywide average and localised hotspots with elevated contamination may occur.

To strengthen future applications of this method, three data priorities are identified. Most critically, nationally consistent depth-to-groundwater measurements are needed. City-level aggregation of sanitary inspection scores for self-supply sources, particularly dug-wells, would directly address one of the largest identified sources of model bias. Characterisation of unsaturated zone properties relevant to microbial attenuation, such as porosity and clay content, would further improve predictive accuracy. Together, these improvements would substantially reduce the uncertainty in city-level risk rankings and increase their utility for investment planning in low- and middle-income urban settings where self-supplied groundwater remains a primary drinking water source.

## Supplementary Information

Below is the link to the electronic supplementary material.

ESM 1 (XLSX 134 KB) 

ESM 2 (DOCX 228 KB)

## Data Availability

The data used for logistic regression is in supplementary information. This dataset is deidentified, providing only the city of each case.

## Footnotes

1 DRASTIC refers to Depth to groundwater; Recharge rate; Aquifer media; Soil media; Topography; Impact of vadose zone; hydraulic Conductivity of the aquifer.

2 GOD refers to Groundwater occurrence, i.e. type of aquifer, Overlying lithology and Depth to groundwater.

3 City defined as government administrative areas known as municipalities or “kota”.

4 Pixel is a rectangle approximately 280 m by 315 m.

## Declarations

All authors have read, understood and complied as applicable with the statement on “Ethical responsibilities of Authors” as found in the Instructions for Authors.
