# Supplementary material for: Ranking microbial risk of self-supplied groundwater in urban Indonesia
Source: Environ Monit Assess. 2026 Sep 23;198(10):1098. doi: 10.1007/s10661-026-15909-7 (PMC13601194; doi:10.1007/s10661-026-15909-7)
Supplement: Supplementary file 1 [file 10661_2026_15909_MOESM2_ESM.docx]

Table S1 Sensitivity Analysis on Sanitary Inspection Score

| **Sanitary Inspection Score** | | **Probability of *E. coli* > 100 CFU/100mL** | | |
| --- | --- | --- | --- | --- |
|  |  | **25%ile of city average for all other parameters** | **50%ile of city average for all other parameters** | **75%ile of city average for all other parameters** |
| minimum | 0 | 0.067 | 0.092 | 0.155 |
| median | 1 | 0.077 | 0.104 | 0.175 |
|  | 2 | 0.087 | 0.118 | 0.196 |
|  | 3 | 0.099 | 0.134 | 0.220 |
| 90%ile | 4 | 0.113 | 0.152 | 0.245 |

Supplementary model with SIS included:

Logit(P) = -3.079 + 0.989 * *S* - 0.504 * *AL* + 0.132 * (*R*/100) + 0.867 * (*PN*/1000) + 0.143 * SIS.	(S1)

where:

*S* = whether the drinking water source is a dug-well (1) or a drilled-bore (0)

*AL* = aquifer lithology is solid or volcanic rock = 1 otherwise 0

*R* = total rainfall from October to November in mm.

*PN* = population per pixel^[1]^.

SIS = Sanitary Inspection Score

Table S2 Cities by risk ranking

| Green = Lowest Risk Tier | Yellow = Middle Risk Tier | Red = highest risk tier |
| --- | --- | --- |

| **City Name** | **Risk Score** | **Risk score lower 95%ile** | **Risk Score Upper 95%ile** | **Tier stable** |
| --- | --- | --- | --- | --- |
| Banjarmasin | 0.000 | 0.000 | 0.001 | TRUE |
| Pontianak | 0.000 | 0.000 | 0.001 | TRUE |
| Samarinda | 0.001 | 0.000 | 0.004 | TRUE |
| Bontang | 0.001 | 0.000 | 0.004 | TRUE |
| Surabaya | 0.002 | 0.001 | 0.006 | TRUE |
| Tanjung Balai | 0.005 | 0.001 | 0.010 | FALSE |
| Tegal | 0.005 | 0.001 | 0.010 | FALSE |
| Banda Aceh | 0.006 | 0.001 | 0.013 | FALSE |
| Denpasar | 0.006 | 0.003 | 0.012 | FALSE |
| Solok | 0.006 | 0.002 | 0.014 | FALSE |
| Jakarta Utara | 0.007 | 0.003 | 0.012 | FALSE |
| Batam | 0.007 | 0.003 | 0.014 | FALSE |
| Jakarta Barat | 0.008 | 0.003 | 0.016 | FALSE |
| Balikpapan | 0.008 | 0.003 | 0.016 | FALSE |
| Madiun | 0.010 | 0.005 | 0.019 | FALSE |
| Jakarta Pusat | 0.010 | 0.005 | 0.020 | FALSE |
| Gorontalo | 0.010 | 0.004 | 0.023 | FALSE |
| Tarakan | 0.010 | 0.004 | 0.022 | FALSE |
| Medan | 0.012 | 0.006 | 0.019 | FALSE |
| Makassar | 0.012 | 0.006 | 0.021 | FALSE |
| Cirebon | 0.013 | 0.006 | 0.022 | FALSE |
| Cilegon | 0.013 | 0.006 | 0.025 | FALSE |
| Payakumbuh | 0.014 | 0.006 | 0.027 | FALSE |
| Palembang | 0.014 | 0.007 | 0.024 | FALSE |
| Sabang | 0.015 | 0.006 | 0.032 | FALSE |
| Pematang Siantar | 0.017 | 0.008 | 0.031 | FALSE |
| Ternate | 0.017 | 0.007 | 0.035 | FALSE |
| Bitung | 0.018 | 0.008 | 0.038 | FALSE |
| Mojokerto | 0.018 | 0.008 | 0.036 | FALSE |
| Pasuruan | 0.020 | 0.009 | 0.042 | FALSE |
| Manado | 0.022 | 0.011 | 0.042 | TRUE |
| Bukittinggi | 0.023 | 0.012 | 0.039 | TRUE |
| Dumai | 0.024 | 0.012 | 0.045 | TRUE |
| Magelang | 0.027 | 0.016 | 0.045 | TRUE |
| Kendari | 0.028 | 0.013 | 0.054 | FALSE |
| Sorong | 0.030 | 0.016 | 0.053 | FALSE |
| Padang Panjang | 0.030 | 0.016 | 0.051 | FALSE |
| Parepare | 0.031 | 0.016 | 0.056 | FALSE |
| Palu | 0.031 | 0.015 | 0.060 | FALSE |
| Tangerang | 0.032 | 0.021 | 0.048 | TRUE |
| Sungai Penuh | 0.032 | 0.016 | 0.058 | FALSE |
| Bekasi | 0.033 | 0.020 | 0.051 | FALSE |
| Probolinggo | 0.033 | 0.015 | 0.067 | FALSE |
| Jakarta Timur | 0.036 | 0.021 | 0.056 | FALSE |
| Salatiga | 0.036 | 0.020 | 0.060 | FALSE |
| Langsa | 0.036 | 0.020 | 0.061 | FALSE |
| Sibolga | 0.036 | 0.020 | 0.058 | FALSE |
| Bandar Lampung | 0.036 | 0.021 | 0.059 | FALSE |
| Mataram | 0.037 | 0.021 | 0.061 | FALSE |
| Serang | 0.038 | 0.021 | 0.066 | FALSE |
| Semarang | 0.039 | 0.025 | 0.059 | FALSE |
| Pekanbaru | 0.039 | 0.021 | 0.068 | FALSE |
| Malang | 0.040 | 0.023 | 0.066 | FALSE |
| Surakarta | 0.042 | 0.027 | 0.063 | FALSE |
| Jayapura | 0.046 | 0.026 | 0.075 | FALSE |
| Sawah Lunto | 0.047 | 0.025 | 0.082 | FALSE |
| Tomohon | 0.047 | 0.022 | 0.094 | FALSE |
| Baubau | 0.047 | 0.023 | 0.084 | FALSE |
| Singkawang | 0.049 | 0.028 | 0.079 | FALSE |
| Baru | 0.050 | 0.027 | 0.085 | FALSE |
| Pangkal Pinang | 0.051 | 0.030 | 0.084 | FALSE |
| Bima | 0.052 | 0.023 | 0.110 | FALSE |
| Kotamobagu | 0.054 | 0.028 | 0.098 | FALSE |
| Palangka Raya | 0.054 | 0.029 | 0.092 | FALSE |
| Lhokseumawe | 0.054 | 0.031 | 0.087 | FALSE |
| Binjai | 0.055 | 0.034 | 0.084 | FALSE |
| Bandung | 0.056 | 0.030 | 0.091 | FALSE |
| Blitar | 0.059 | 0.032 | 0.104 | FALSE |
| Palopo | 0.061 | 0.034 | 0.097 | FALSE |
| Tebing Tinggi | 0.062 | 0.037 | 0.100 | FALSE |
| Tanjung Pinang | 0.062 | 0.033 | 0.109 | FALSE |
| Kediri | 0.064 | 0.035 | 0.113 | FALSE |
| Kupang | 0.065 | 0.032 | 0.122 | FALSE |
| Jakarta Selatan | 0.069 | 0.045 | 0.101 | FALSE |
| Tual | 0.070 | 0.036 | 0.126 | FALSE |
| Banjar Baru | 0.070 | 0.039 | 0.117 | FALSE |
| Tangerang Selatan | 0.078 | 0.053 | 0.110 | TRUE |
| Pekalongan | 0.080 | 0.050 | 0.125 | FALSE |
| Padang | 0.081 | 0.048 | 0.125 | FALSE |
| Bogor | 0.082 | 0.047 | 0.132 | FALSE |
| Cimahi | 0.084 | 0.053 | 0.125 | TRUE |
| Depok | 0.084 | 0.056 | 0.122 | TRUE |
| Jambi | 0.085 | 0.056 | 0.120 | TRUE |
| Tidore Kepulauan | 0.095 | 0.048 | 0.173 | FALSE |
| Yogyakarta | 0.095 | 0.064 | 0.138 | TRUE |
| Metro | 0.103 | 0.058 | 0.173 | TRUE |
| Batu | 0.107 | 0.063 | 0.176 | TRUE |
| Sukabumi | 0.111 | 0.065 | 0.174 | TRUE |
| Banjar | 0.118 | 0.069 | 0.186 | TRUE |
| Bengkulu | 0.122 | 0.066 | 0.203 | TRUE |
| Tasikmalaya | 0.131 | 0.069 | 0.221 | TRUE |
| Pariaman | 0.142 | 0.075 | 0.241 | TRUE |
| Padangsidimpuan | 0.147 | 0.090 | 0.225 | TRUE |
| Ambon | 0.162 | 0.097 | 0.244 | TRUE |
| Subulussalam | 0.166 | 0.103 | 0.254 | TRUE |
| Prabumulih | 0.175 | 0.101 | 0.280 | TRUE |
| Gunungsitoli | 0.182 | 0.119 | 0.258 | TRUE |
| Lubuklinggau | 0.213 | 0.124 | 0.336 | TRUE |
| Pagar Alam | 0.227 | 0.135 | 0.357 | TRUE |

Figure S1 Model Calibration Plot

1. Pixel is a rectangle approximately 280 m by 315 m
